# A new species of *Notodiaptomus* from the Ecuadorian Andes (Copepoda, Calanoida, Diaptomidae)

**DOI:** 10.3897/zookeys.697.12204

**Published:** 2017-09-14

**Authors:** Miguel Alonso, Edinaldo N. dos Santos-Silva, Damià Jaume

**Affiliations:** 1 Departamento de Recursos Hídricos y Ciencias Ambientales, Facultad de Ciencias Químicas, Universidad de Cuenca, Cuenca, Ecuador; 2 Ecology Section, Department of Evolutionary Biology, Ecology and Environmental Sciences, Faculty of Biology, University of Barcelona, Avda. Diagonal 643, 08028 Barcelona, Spain; 3 Laboratório de Plâncton, Instituto Nacional de Pesquisas da Amazônia. Coordenação de Pesquisas em Biodiversidade, Av. André Araújo 2936, 69060-001 Manaus, Amazonas, Brazil; 4 IMEDEA (CSIC-UIB), Mediterranean Institute for Advanced Studies, C/ Miquel Marquès 21, 07190 Esporles, Illes Balears, Spain

**Keywords:** Crustacea, Ecuador, reservoirs, South America, zooplankton

## Abstract

*Notodiaptomus cannarensis* **sp. n.** is described from a reservoir on the Amazonian slope of the Ecuadorian Andes. The new species is unique among diaptomid calanoid copepods in the display of hypertrophied, symmetrical wing-like extensions at each side of the female composite genital somite. Furthermore, it displays a female urosome reduced to only two somites due to the incorporation of abdominal somites III and IV to the composite genital double-somite, and a male right fifth leg with the outer spine of second exopodal segment recurved and implanted proximally on margin. It differs from any other *Notodiaptomus* in the display of a large rectangular lamella on proximal segment of exopod of male right fifth leg. The species is currently known only from Mazar reservoir, a eutrophic water body placed above 2127 m a.s.l. on the River Paute (Cañar Province; southern Ecuador), where it is the most common crustacean in the water column.

## Introduction

The inland waters of the Neotropical region harbour representatives of at least three different calanoid copepod families, *viz.* Centropagidae Giesbrecht, 1892, Pseudodiaptomidae G.O. Sars, 1902, and Diaptomidae Baird, 1850. The Centropagidae (22 species reported thus far; Boxshall and Defaye 2008) are distributed from Patagonia to the Andes, with only three species known to occur out of those regions, in SE Brazil (Previattelli et al. 2013). The Pseudodiaptomidae appear mainly in estuaries and other shallow coastal marine habitats of reduced salinity, and include notorious examples of accidental translocation of exotic species (Andrade dos Santos et al. 2009); four native species of *Pseudodiaptomus* Herrick, 1884 have been reported so far from the region under consideration (Santos-Silva 2008). Finally, the Diaptomidae are distributed through the rest of South America except at high altitudes and latitudes, with a broad overlapping zone with the area exclusive of the Centropagidae embracing from 28°S (Santa Catarina State, Brazil) to almost 39°S in the Argentinian northern Patagonia (Bănărescu 1990; Previattelli et al. 2015). The most recent accounts (Perbiche-Neves et al. 2014) estimate in 98 the number of species of diaptomids known from the Neotropical region, distributed over 15 genera, 14 of which being endemic (Dussart and Defaye 2002). Here we describe a new species of diaptomid of the genus *Notodiaptomus* Kiefer, 1936, from the Amazonas High Andes Ecoregion (*sensu* Abell et al. 2008).

## Materials and methods

The copepods were collected in the water column of Mazar reservoir using a plankton net of 60 µm mesh size hauled from 25 m depth. Sampling was performed in the framework of the project “Comprensión de los Procesos Hidroecológicos como base para la Estimación del Caudal Ecológico en las Cuencas del Jubones y Paute”, sponsored by the Secretaría Nacional de Educación Superior, Ciencia, Tecnología e Innovación of the Government of the Republic of Ecuador. Water in the reservoir is poorly mineralized (56-62 µS/cm) and turbid due to presence of phytoplankton (Secchi Disk depth 1.4 m), in accord to its eutrophic condition. Material was fixed *in situ* with formalin and dissected in glycerine on an excavated slide. Drawings were prepared with a camera lucida attached to an Olympus BH-2 microscope equipped with phase contrast. Terminology used in descriptions follows Huys and Boxshall (1991). Type material is deposited in the Museo Ecuatoriano de Ciencias Naturales del Instituto Nacional de Biodiversidad, Quito, Ecuador [MECN].

## Taxonomy

### Subclass COPEPODA Milne Edwards, 1830

#### Order CALANOIDA Sars, 1903

##### Family DIAPTOMIDAE Baird, 1850

###### Genus *Notodiaptomus* Kiefer, 1936, emend. Santos-Silva, Boxshall & da Rocha, 1999

####### Notodiaptomus cannarensis sp. n.

Taxon classificationAnimaliaCalanoidaDiaptomidae

http://zoobank.org/4D4FEBB1-4A88-4BD8-8593-2EAD177E0956

**Figure 1.**
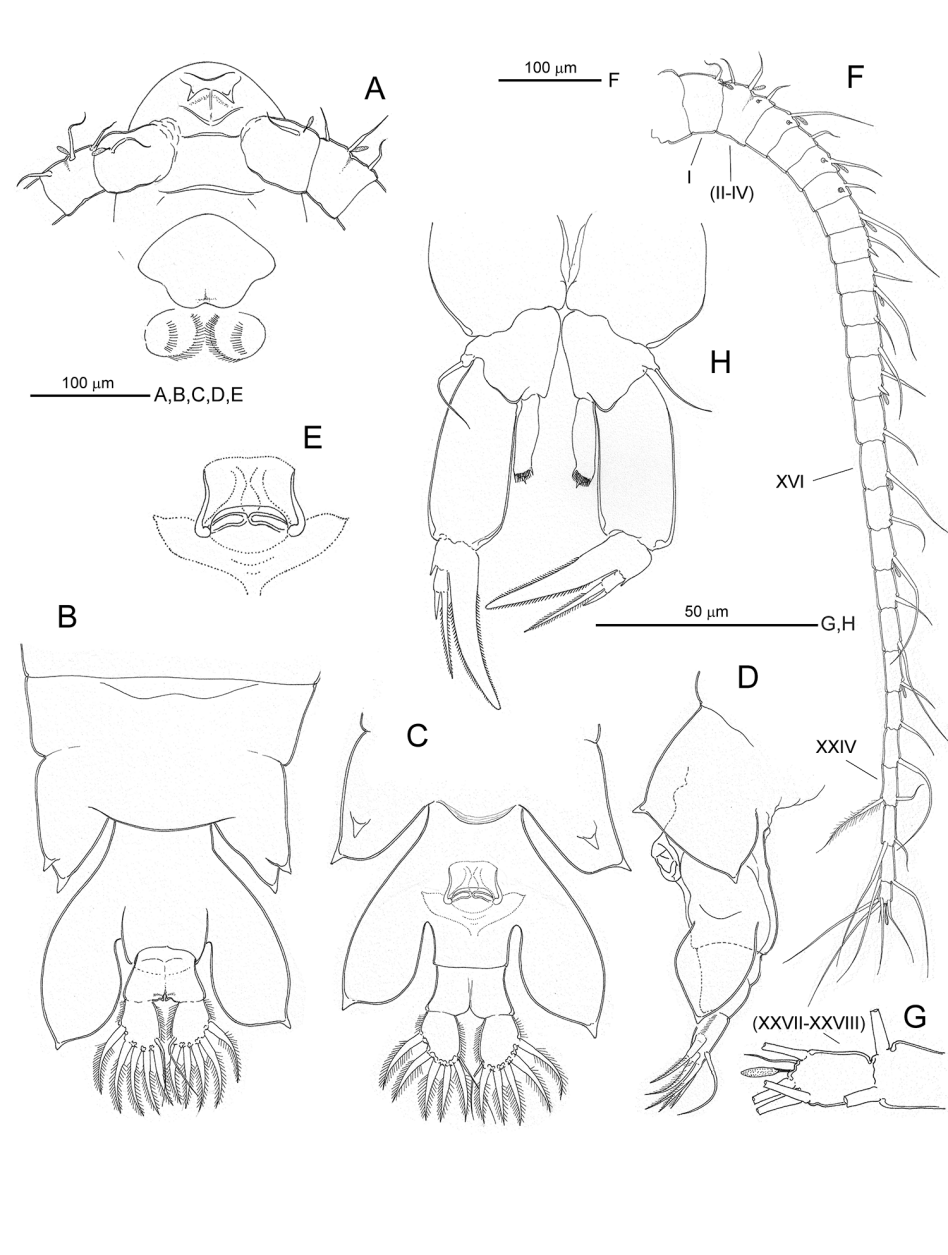
*Notodiaptomus cannarensis* sp. n., adult female. **A** anterior portion of prosome showing rostrum, insertion of antennules, labrum and paragnaths, ventral **B** last pedigerous somite plus urosome and caudal rami, dorsal **C** same, ventral **D** same, left lateral **E** inset of genital aperture **F** right antennule, ventral **G** inset of terminal segments of latter **H** fifth legs, posterior.

**Figure 2.**
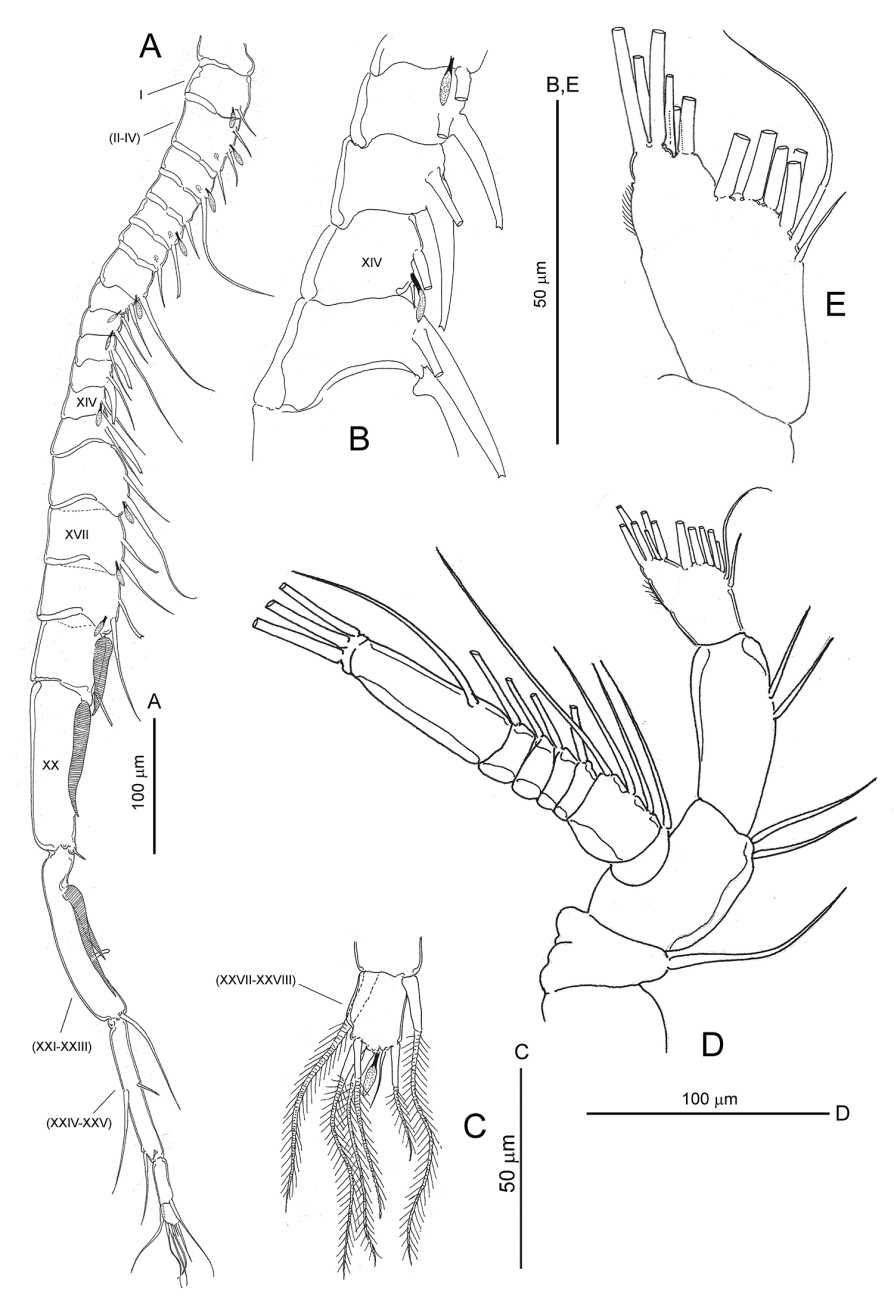
*Notodiaptomus cannarensis* sp. n. **A** male right antennule, ventral **B** detail of segments 10 to 13 of latter **C** inset of terminal segment **D** adult female right antenna, ventral **E** inset of terminal segment of latter.

**Figure 3.**
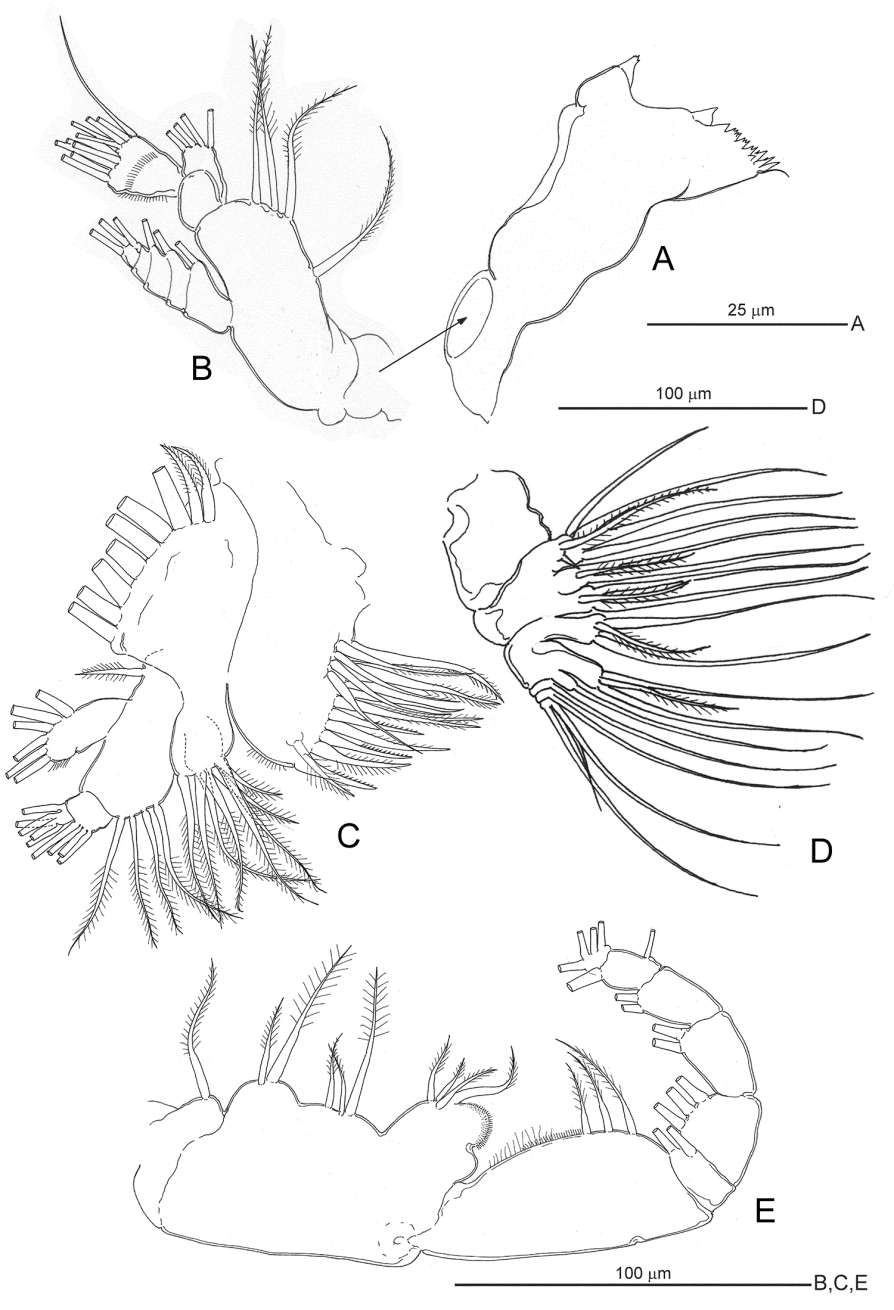
*Notodiaptomus cannarensis* sp. n., adult female. **A** mandible coxal gnathobase **B** mandibular palp **C** maxillule **D** maxilla **E** maxilliped.

**Figure 4.**
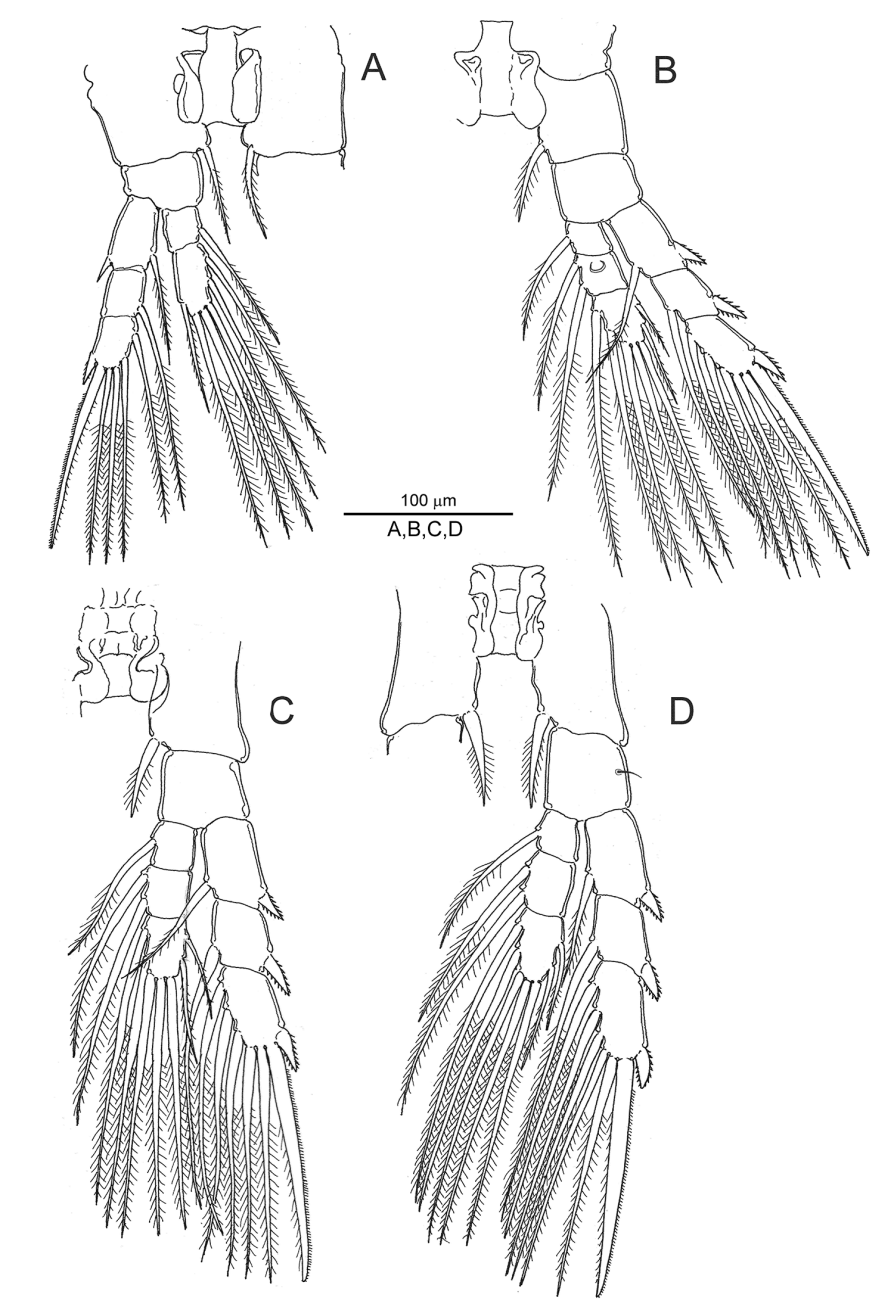
*Notodiaptomus cannarensis* sp. n., adult female. **A** left leg 1, posterior view **B** right leg 2, posterior **C** left leg 3, anterior **D** right leg 4, anterior.

**Figure 5.**
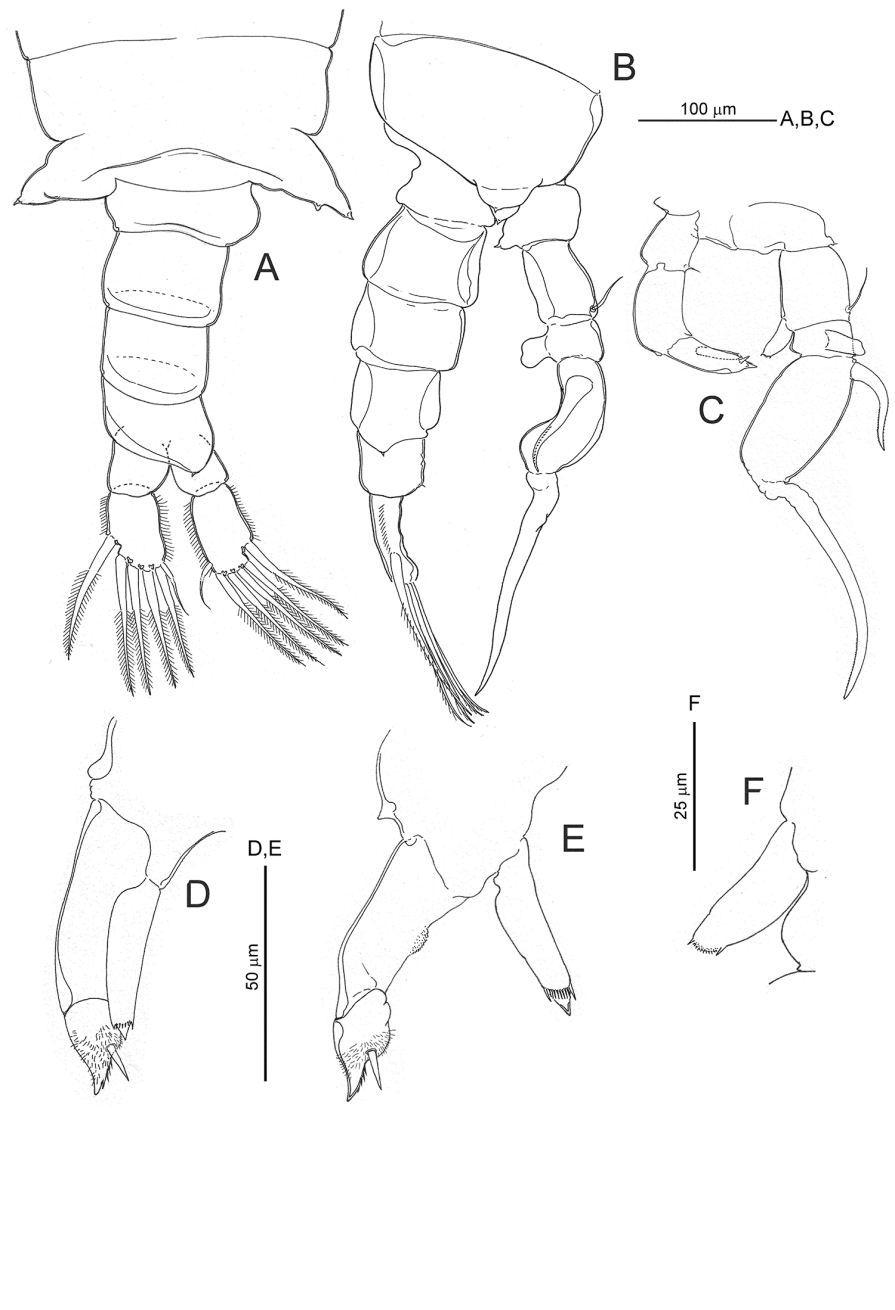
*Notodiaptomus cannarensis* sp. n., adult male. **A** last pedigerous somite plus urosome and caudal rami, dorsal **B** inset of last pedigerous somite with fifth legs attached plus urosome and caudal rami, right lateral aspect **C** fifth legs, posterior **D** and **E** different aspects of left fifth leg rami **F** inset of right fifth leg endopod.

Figs 1 , 2 , 3 , 4 , 5

######## Material examined.

Mazar reservoir (River Paute, Cañar Province, southern Ecuador). Coordinates 2°35’53.08”S; 78°37’32.16”W. Altitude: 2127 m a.s.l. Holotype: male 1.2 mm long, preserved in formalin vial. Paratypes: Ten males and ten females, preserved in formalin vial. Holotype and paratypes registered under same registration number [MECN-SI-Cal-0001]. Collected by Verónica Ordóñez, April 2013.

######## Diagnosis.

Female urosome reduced to only two somites due to incorporation of abdominal somites III and IV into composite genital double-somite; resulting composite somite with symmetrical, hypertrophied wing-like (in dorsal aspect) extensions at each side. Male right fifth leg with outer spine of second exopodal segment recurved and implanted proximally on margin.

######## Etymology.

Species name refers to the Ecuadorian province where it was found (Cañar Province; southern Ecuador).

######## Distribution.

Known only from Mazar reservoir, located on the River Paute (Amazon Basin, Cañar Province, southern Ecuador), 2127 m a.s.l.

######## Description of adult female.

Body up to 1.4 mm long. *Prosome* 5-segmented, comprising cephalosome plus first to third free pedigerous somites, and partially-fused fourth and fifth pedigerous somites (Fig. 1B–D); epimeral plates of latter extended backwards and displaying two pointed processes at each side, oriented as figured. *Rostrum* (Fig. 1A) bifid, with paired short rostral filaments. *Urosome* (Fig. 1B–D) 2-segmented, with genital somite incorporating all abdominal somites except anal somite; resulting composite genital somite displaying pair of hypertrophied ventrolateral ovoid swellings extended backwards, each with pointed tip. Genital field not fully resolved, with paired gonoporal plates placed medially on ventral surface of composite genital somite, partially covered with short genital operculum (Fig. 1E). Caudal rami symmetrical, slightly longer than broad with setulose margins; caudal setae symmetrical, short, all plumose except dorsal seta, simple and more slender than rest; anterolateral accessory seta absent.

*Antennules* (Fig. 1F, G) symmetrical, each 25-segmented, with fusions affecting ancestral segments II-IV and XXVII-XXVIII; segmentation pattern and armature formula as follows: segment 1 (corresponding to ancestral segment I), 1 seta + aesthetasc; segment 2 (fused ancestral segments II-IV), 3 setae + aesthetasc; segment 3 (V), 1 seta + ae; segment 4 (VI); 1 seta; segment 5 (VII), 1 seta + ae; segment 6 (VIII), 1 seta; segment 7 (IX), 1 seta + ae; segment 8 (X), 2 setae, of which distal most reduced; segment 9 (XI), 2 setae + ae; segments 10 (XII) and 11 (XIII), 1 seta each; segment 12 (XIV), 2 setae + ae, with distal seta reduced; segment 13 (XV), 1 seta; segment 14 (XVI), 1 seta + ae; segment 15 (XVII), 1 seta; segment 16 (XVIII), 1 seta+ ae; segments 17 (XIX) and 18 (XX), 1 seta each; segment 19 (XXI), 1 seta+ ae; segments 20 (XXII) and 21 (XXIII), 1 seta each; segments 22 (XXIV) to 24 (XXVI), 1 + 1 setae each; segment 25 (fused XXVII-XXVIII), 5 setae + ae. Sensilla present on anterodorsal surface of segments 2, 3, 5 and 6.

*Antenna* (Fig. 2D, E) biramous. Coxa with one seta on medial margin. Basis with two setae on distomedial margin. Exopod 8-segmented, setal formula 1, 3, 1, 1, 1, 1, 1, 3. Endopod 2-segmented with compound second segment bilobed; setal formula 2, 7+6 (see Fig. 2E).

*Labrum* (Fig. 1A) with concave distal margin. *Paragnaths* (Fig. 1A) globose, each with two rows of setae as figured.

*Mandible* coxal gnathobase (Fig. 3A) cutting edge 9-denticulate and with simple distal seta; innermost denticle broadly separated from rest. Palp (Fig. 3B) biramous, basis with four medial setae; exopod 5-segmented, setal formula 1, 1, 1, 1, 2; endopod 2-segmented, distal segment bilobed, with row of setules along outer margin and transverse row of setules about midway, setal formula 4, 5+5.

*Maxillule* (Fig. 3C) praecoxal arthrite with 15 armature elements ornamented and distributed as figured. Coxal epipodite with nine setae; coxal endite with four setae. Basal exite seta present; basal endites each with four setae. Exopod with six setae. Endopod 2-segmented, setal formula 4, 5.

*Maxilla* (Fig. 3D) syncoxal endites armature formula: 5 + reduced spine, 3, 3, 3. Allobasis basal endite with three setae; allobasis endopodal endite with single seta. Free endopod 3-segmented, setal formula 1, 1, 3.

*Maxilliped* (Fig. 3E) praecoxal endite with single seta. Coxal endites with 2, 3, and 4 setae, respectively; distal endite with spinulose swelling. Basis with three medial setae. Endopod 6-segmented, armature formula 2, 3, 2, 2, 1+1, 4.

*Swimming legs* 1-4 (Fig. 4) each biramous with 3-segmented rami except for 2-segmented endopod on leg 1 (Fig. 4A). Second endopodal segment of leg 2 with smooth rounded swelling (“Schmeil’s organ”) on posterior surface (Fig. 4B). Outer exopodal spines reduced in all limbs. Armature formula as follows:

**Table:** 

	Coxa	Basis	Exopod	Endopod
Leg 1	0-1	0-0	I-1; 0-1; I,I,4	0-1; 1,2,3
Legs 2 & 3	0-1	0-0	I-1; I-1; I,I,5	0-1; 0-2; 2,2,3
Leg 4	0-1	1-0	I-1; I-1; I,I,5	0-1; 0-2; 2,2,3

*Fifth legs* (Fig. 1H) symmetrical, biramous, coxa and basis separate, outer basal seta simple and implanted on socle. Exopod 3-segmented, proximal segment unarmed, longer than middle segment; distomedial angle of middle segment prolonged into stout spinous process fringed with short setules, distolateral angle with tiny smooth spinous process; distal segment reduced, with two unequal setae distally, innermost longer and setulose, but not as long as inner spinous process of middle segment–, outermost seta much shorter and smooth. Endopod unsegmented, subrectangular with three tiny spinous processes and transverse row of setules distally.

######## Description of male.

Body up to 1.22 mm long. Differing from female in fifth legs, modified right antennule, asymmetrical epimeral plates of composite last prosomal somite, and segmentation and asymmetry of urosome, including caudal rami. Thus, the extensions of the epimeral plates corresponding to the partially fused fourth and fifth pedigerous somites are directed laterally instead of backwards, with the pointed processes present on each side less marked than in the female (Fig. 5A, B). The *urosome* is 5-segmented, with the genital somite asymmetrical, slightly protruding on the right side; the third abdominal somite is also asymmetrical, showing a dorsolateral hump crowned with a tiny spine on the right side. In addition, the *caudal rami* are slightly asymmetrical and comparatively more elongated than in female, with proportionally longer caudal setae except for dorsal seta, that is shorter.

*Right antennule* (Fig. 2A–C) 22-segmented, geniculate, with geniculation located between segments 18 (corresponding to ancestral segment XX) and 19 (corresponding to fused ancestral segments XXI-XXIII). Other fusions involving ancestral segments II-IV, XXIV-XXV and XXVII-XXVIII. Segmentation pattern and armature formula as follows: segment 1 (ancestral I), 1 seta + aesthetasc; segment 2 (II-IV), 3 setae + ae; segment 3 (V), 1 seta + ae; segment 4 (VI), 1 seta; segment 5 (VII), 1 seta + ae; segment 6 (VIII), 1 seta; segment 7 (IX), 1 seta + ae; segment 8 (X), 2 setae, of which distal reduced, conical; segment 9 (XI), 2 setae + ae; segments 10 (XII) and 11 (XIII), each with 1 stout truncate spiniform process + 1 seta; segment 12 (XIV), 2 setae + ae, with distal seta reduced, conical; segment 13 (XV), 1 stout truncate spiniform process plus seta; segment 14 (XVI), 2 setae + ae; segments 15 (XVII) and 16 (XVIII), each with 1 slender truncate spiniform process proximally and 1 seta + ae distally; segment 17 (XIX), 1 striated hyaline seta proximally + reduced truncate spiniform process distally; segment 18 (XX), 1 striated hyaline seta proximally plus tiny seta distally; segment 19 (XXI-XXIII), 2 striated hyaline setae plus short blunt seta midway of margin plus distal seta; segment 20 (XXIV-XXV), 2 + 2 setae; segment 21 (XVI), 1 + 1 setae; segment 22 (XVII-XXVIII), 5 setae + ae. Segments 13 to 18 swollen. Anterodorsal sensilla present on each segments 2, 3, 5 and 6.

*Fifth leg* (Fig. 5C) biramous, highly asymmetrical, each with coxa fused to intercoxal sclerite. Coxa and basis separate on both sides, each with tiny posterolateral seta implanted on socle. Right leg largest, basis with simple seta on posterolateral margin; Exopod 3-segmented, proximal segment with large rectangular lamella implanted on posterior surface (see Fig. 5B, C), second segment expanded and armed with recurved spine proximally on outer margin; distal segment of exopod modified as curved claw with inner margin finely serrated distally. Endopod (Fig. 5F) unsegmented, short –not surpassing distal margin of proximal exopodal segment–, subrectangular with tiny terminal spine on each side; distal margin of segment finely denticulate. Left leg (Fig. 5D, E) basis unarmed. Exopod 2-segmented, distal segment shortest, bifid with distal spine and subdistal conical robust seta, densely covered with short setules; proximal segment with micro-denticulate rounded outgrowth about midway of inner margin. Endopod unsegmented, implanted on produced distomedial angle of basis, with pointed tip wearing tiny spine subdistally at each side plus transverse row of setules in between as figured.

## Remarks

The new species described herein corresponds in almost all respects to the re-diagnosis of *Notodiaptomus* Kiefer, 1936, as presented by Santos-Silva et al. (1999) and Previatelli (2010). This is the most broadly distributed and species-rich genus of freshwater calanoids in the Neotropics, embracing currently 39 nominal species (Santos-Silva 2008).

The new taxon can be distinguished from any other representative of the genus by its female composite genital somite, which displays a hypertrophied, wing-like (in dorsal aspect) extension at each side; no other calanoid copepod is known to display such hypertrophied symmetrical extensions, although two Neotropical taxa, viz. *Tumeodiaptomus* Dussart, 1979, and some members of *Rhacodiaptomus* Kiefer, 1936 (e.g. *Rhacodiaptomus besti* Santos-Silva & Robertson, 1993, and in a lesser extent *R. insolitus* (Wright, 1927) or *R. retroflexus* Brandorff, 1973), show somewhat similar structures but with only one of the two wings hypertrophied (see Dussart 1979; Santos-Silva and Robertson 1993). Nevertheless, at least *Tumeodiaptomus* appears distantly related to *Notodiaptomus* in Previatelli’s (2010) analysis of the phylogenetic relationships of Neotropical diaptomids. In addition, the female urosome reduced to only two somites, and the proximal placement of the outer spine on margin of the second exopodal segment of the male right fifth leg are also salient traits of the new taxon. Nevertheless, a 2-segmented condition of the female urosome is known also to occur in several non-Neotropical diaptomids such as in many species of *Tropodiaptomus* Kiefer, 1932, and several *Thermodiaptomus* Kiefer, 1932 and *Mixodiaptomus* Kiefer, 1932. On the other hand, males of *Tumeodiaptomus* show also the outer spine on margin of the second exopodal segment of the right fifth leg inserted proximally, but not as close to the base of the segment as in the new species.

Apart from these unique features, the new species shows a series of character states in the armature of several limbs that differ from the condition found in the type-species of the genus *N. deitersi* (Poppe, 1891) as redescribed by Santos-Silva et al. (1999). Thus, the distal segment of the antennules of both sexes displays five setae plus aesthetasc (versus 4 + ae in *N. deitersi*); there is a sensilla present on antennulary segments 2, 3, 5 and 6 (versus sensilla present only on segments 2, 3 and 5 in *N. deitersi*); segments 15 and 16 of the geniculate male antennule are devoid of pointed process (versus process present on both segments in *N. deitersi*); segments 17 and 18 are each armed with a modified hyaline seta and a short ordinary setae (versus modified hyaline seta + 2 ordinary setae present on each in *N. deitersi*); the distal endopodal segment of the mandibular palp bears ten setae (versus only nine in *N. deitersi*); the proximal endopodal segment of maxilla displays four setae (versus only three in *N. deitersi*); and the distal coxal endite of maxilliped displays four setae (versus three in *N. deitersi*). Both taxa differ notoriously also in the ornamentation of the rounded outgrowth present on the medial margin of the proximal exopodal segment of male left fifth leg (micro-denticulate in the new species, versus covered with long setules in *N. deitersi*); in the relative length and outline of endopod of male fifth leg (subrectangular and longer than the corresponding proximal exopodal segment in the new species, versus subtriangular and shorter than proximal exopodal segment in *N. deitersi*); and in the relative size of the distolateral spine of the second exopodal segment of the female fifth leg (shorter than the third segment in the new species, versus as long as segment in *N. deitersi*).

The new species differs also from any other *Notodiaptomus* in the display of a large rectangular lamella on proximal segment of exopod of male right fifth leg.

## Supplementary Material

XML Treatment for Notodiaptomus cannarensis
